# 

**Published:** 1851-01

**Authors:** 


### CONTENTS:

### PART I.—ORIGINAL COMMUNICATIONS.

ART. 1.—Crural Phlebitis—by David Hutchinson, M. D. - - - p.339

### PART II.—REVIEWS AND NOTICES OF NEW WORKS.

ART. 1.—Report of Committee on Medical Sciences—By A. B. Palmer, M. D. - - - - - - - - - 355
" 2.—Report of Committee on Practical Medicine—By J. M. Evans, M. D. - - - - - - - - - 372
" 3.—Southern Medical Reports—By N. S. Davis, M. D. - - 385
" 4.—Materia Medica, and Therapeutics—By Thomas D. Mitchell, A. M., M. D. - - - - - - - 345
" 5.—Causes, Nature and Treatment of Palsey and Apoplexy—By James Copeland, M. D., F. R. S. - - - - - 449
" 6.—Researches upon the Necropolis of New Orleans—By Bennett Dowler, M. D. - . - - - - 550
" 7.—Comparative liability of Males and Females to Insanity—By Edward Jarvis, M. D. - - - - - - 551
" 8.—Human Physiology—By Robley Dunglison, M. D. - - 352
" 9.—Diseases and Physical Education of Children—By John Eberle, M. D. - - - - - - · - 353
" 10.—Anatomy, Physiology, Hygiene, etc.—By Calvin Cutter, M. D. 354

### PART III.—SELECTIONS.

ART. 1.—Has the Cerebellum any special connection with the sexual propensity of Function of Generation—By N. S. Davis, M. D. 390
" 2.—Ergotine in External and Internal Hemorrhages—By J. M. Bonjean. 393
" 3.—Vaginal and Uterine Discharges—By Thomas W. Cooke, Esq. 395
" 4.—Cotton Seed in Intermittant Fever - - - 396
" 5.—Collodion in Small Pox—By S. B. Brinckerhoff, M. D. - - 397
" 6.—Prof. Austin Flint's Card. . - - - 298
" 7.—Prof. Eve's Card. 299
" 8.—Operative Surgery—By Prof. Mott. - - - 400
" 9.—Medical Gleanings in Naples. , - - - 401
" 10.—Singular Revenge. - - - - - 402
" 11.—Iodine in Snake Bite. - - - - 403
" 12.—Cutaneous Poison, - - - , - 404
" 13.—Re-Vaccination in Prussia· - - - - 404
" 14.—Attending Families by the Year. - - - 405

### PART IV.—EDITORIAL.

ART. 1.—The Physician and Patient. - - - - - 406
" 2.—Our Journal. - - - - - 407
" 3.—Dr. Davis' History. - - - - - 408
Notes to Readers and Correspondents. -, - - - - 409

